# The influence of different levels of physical activity and sports performance on the accuracy of dynamic lower limbs balance assessment among Chinese physical education college students

**DOI:** 10.3389/fphys.2023.1184340

**Published:** 2023-06-21

**Authors:** Xuejuan Huang, Zhiyang Yan, Yong Ma, Hua Liu

**Affiliations:** ^1^ Department of Sports Engineering and Information Technology, Wuhan Sports University, Wuhan, China; ^2^ Research Center of Sports Equipment Engineering Technology of Hubei Province, Wuhan Sports University, Wuhan, China; ^3^ Department of Sports Medicine, Wuhan Sports University, Wuhan, China

**Keywords:** dynamic lower limbs balance, y balance test, physical activity, sports performance, sports injury risk

## Abstract

**Background:** Balance ability is the basis of human actions. Improving the accuracy of dynamic balance assessment can increase the efficiency of sports injury prediction.

**Objectives:** This study aimed to investigate how physical activity and sports performance affect the dynamic balance ability of lower limbs and validate whether the Lower Quarter Y-Balance Test (YBT-LQ) is a reliable predictor of sports injury risk among Chinese physical education college students.

**Materials and Methods:** In total, 169 voluntary participants completed the YBT-LQ at the beginning of a semester and provided some physiological information and an injury report at the end of the semester. The correlation between YBT-LQ performance and selected factors that can affect the dynamic balance control was analyzed based on data statistics. The receiver operating characteristic (ROC) and the area under curve (AUC) of the composite scores of the YBT-LQ were calculated to explore an optimal cutoff value for predicting sports injury risk.

**Results:** The composite scores of the YBT-LQ exhibited strong correlations with both the sports performance level and sports injury, as well as a moderate correlation with physical activity level, age (negative), and metabolic equivalent (MET). In the entire study population, the area under the receiver operating characteristic (ROC) curves for the binary classification of composite YBT-LQ scores of the left and right legs to predict sports injury risk were 0.78 and 0.74, respectively. Stratifying the study participants based on their levels of physical activity and sports performance had an effect on the AUC values of ROC curves. The optimal cutoff scores of the YBT-LQ for predicting sports injury risk were variable, with values more or less than 95%. Specifically, the cutoff scores for participants with the highest level of sports performance were notably higher, reaching up to 106.5% (left) and 107.2% (right).

**Conclusion:** Physical activity and sports performance can influence human dynamic balance control. Composite scores of the YBT-LQ can be used with acceptable efficiency to predict sports injury. Stratifying participants based on their levels of physical activity and sports performance leads to different optimal cutoff values of the YBT-LQ composite scores in predicting sports injury. This approach is preferable to relying solely on a uniform 95% cutoff. It is recommended to analyze individuals with higher levels of sports performance, such as elite athletes, separately from those with lower levels. This is because the former group has a higher optimal cutoff value compared to the latter.

## 1 Introduction

Balance ability is the basis of all safe movement. Bad balance ability leads to an increase in the risk of falling and harms human physical health. To improve health by avoiding sports injuries during exercises, some assessments need to be studied in advance. The Star Excursion Balance Test (hereafter SEBT) is one of the most commonly used methods for measuring dynamic postural control capability (Kinzey and Armstrong, 1998) (Gribble et al., 2012a). Based on a simplification of the SEBT, the Y-Balance Test (hereafter YBT) was developed as a kind of test to evaluate dynamic balance ability, functional symmetry, and injury risk of limbs with the advantages of easy operation, a shorter duration, and a more standardized process (Coughlan et al., 2012) (Su et al., 2021). According to the target part of the body, the YBT includes two tests: the Upper Quarter Y-Balance Test (hereafter YBT-UQ) and the Lower Quarter Y-Balance Test (hereafter YBT-LQ), representing the YBT test for upper and lower limbs, respectively.

Is YBT performance an effective predictor of sports injury? The current studies show polarization. One study pointed out that high YBT-LQ posteromedial asymmetry is associated with an increased injury risk for elite adolescent Australian footballers, especially for athletes with good agility performance, which could provide a suggestion for athlete preparation programs (Bennett et al., 2022). It was demonstrated that the sports injuries of Chinese firefighters could be singly predicted by YBT performance, but the prediction accuracy could be improved by combining it with the Functional Movement Screen (FMS) (Yunke et al., 2022), another predictive functional screening tool. However, other studies have questioned the YBT as a predictor for sports injuries. For example, one study explained that the performance of the YBT could not be used to predict injury risk in runners because the composite scores of the YBT could not predict 12 variables linked to running injuries according to the linear regression results (Scott and James, 2019). In another study, the YBT was argued as a sole screening method to identify those at risk of lower extremity injuries in Gaelic games; however, it is useful that the YBT could identify those not at risk of non-contact injury. Additionally, the study reminded us that the cut-off points should be analyzed in combination with the specified sports (OConnor et al., 2020).

This study aimed to investigate whether the combination of test performance and relevant factors of dynamic balance ability can enhance the effectiveness of the YBT as a predictor of sports injury. What factors could affect the dynamic balance ability of the human body? Except for genetic heritage and other inherent health states, physical activity (hereafter PA) is one of the essential factors that should be taken into account. The WHO defines physical activity as any bodily movement produced by skeletal muscles that requires energy expenditure. Regular physical activity can improve muscular and cardiorespiratory disease, bone and functional health, and reduce the risk of cardiovascular disease, cancer, and falls, as well as hip or vertebral fractures (team, 2022). In addition, the benefits and risks of exercise, which refers to planned and purposeful physical activity, can also influence the dynamic balance ability of humans. Therefore, sports performance is the other factor taken into account in the study. Sports performance (hereafter SP) is the manner in which sport participation is measured and is the complex mixture of biomechanical function, emotional factors, and training techniques. Better sports performance means a higher athletic ability in the specified sport (of Sports Medicine, 2013).

A study found that subjects with a lack of sports participation or low levels of physical activity had a higher risk of injury (ESL et al., 2017). There is great difference in balance ability among athletes from different sports, with gymnasts having the best ability, followed by soccer players, swimmers, and basketball players (Hrysomallis, 2011). However, sports majors scored higher YBT composite scores than ordinary college students in both the overall sample and the female sample, while there was no significant difference in the male sample (Zhenzhong and Jin, 2019). There are large individual differences in daily activities due to variations in social roles and environments among individuals. The levels of physical activity can be identified through some quantitative means such as by calculating metabolic equivalents. On the other hand, the level of sports performance of a person could be determined by judging his or her professional physical training experience. Therefore, is it appropriate to use the same threshold for dynamic balance assessment to predict sports injuries among populations with varying levels of physical activity and sports performance? Is it essential to stratify participants before predicting sports injuries based on dynamic lower limbs balance assessment? It remains currently unclear whether physical activity and sports performance can influence the accuracy of the YBT-LQ as a dynamic lower balance assessment tool in predicting sports injuries. The study aimed to investigate the effect of physical activity and sports performance on the dynamic balance ability of the lower limbs and validate the reliability of the YBT-LQ as a predictor of sports injury risk among Chinese physical education college students.

## 2 Materials and methods

### 2.1 Participants

A total of 169 college students were selected based on convenience sampling as volunteers for the study. They were all recruited from Wuhan Sports University in Central China, and included 98 males and 71 females. The age range of subjects was 15–24 years old. They were all sophomores in the college.

There were two administrators involved in the tests in the study, whose responsibility was to ensure the procedure of the tests according to the protocol. These administrators were qualified physical fitness coaches who had at least 2 years of experience in physical function tests, such as the FMS, YBT, and so on. One administrator oversaw the leg length measurements while the other guided the stretching measurements in three directions.

### 2.2 Protocol

#### 2.2.1 Dynamic lower limbs balance assessment

The dynamic balance of the lower limbs was measured through the YBT-LQ in three directions (anterior, posteromedial, and posterolateral) for each leg separately (Almeida et al., 2017). At the beginning of the YBT-LQ, the length of the lower limbs of each subject, the distance from the anterior superior iliac spine to the midpoint of the medial malleolus of the same foot, was measured and recorded after he or she took off his or her shoes and socks. The test starting position of the subject was to stand on the test platform with one foot, align the big toe with the red starting line on the test platform, and put both hands on their hips. During the test, the subjects pushed the test panel forward, back inside, and back outside with the other foot except for standing support, and then returned to the starting line. The farthest distance of pushing the test panel in different directions were recorded. The subjects exchanged feet to support standing, repeated the above standardized test process three times, and the test results were recorded, respectively.

If the following conditions occurred, the test would be considered invalid and needed to be restarted (Plisky et al., 2006). Firstly, if the subject’s body lost balance during the test, for example, the support leg moved, the heel was lifted or left the center plate, or the hand touched the ground or the test panel to obtain support during the tests. Secondly, if the subject used the freely movable foot to step on the test board or the test rod as a strength source for supporting the body, or his or her feet touched the ground in either test direction. Thirdly, if the subject kicked or pushed the indicator forcefully to make the indicator slide forward with the aid of inertia to obtain a longer extension distance. Finally, if the starting body posture of the subject was not consistent with the ending posture.

#### 2.2.2 Normalization of YBT-LQ score calculation

The YBT showed excellent reliability in the anterior, posterior lateral, and posterior medial direction as a measure of postural stability (Almeida et al., 2017). Each action in three directions was allowed to be repeated three times in the specified mode. The maximum reach of each action in three directions was recorded to calculate the composite score of the YBT-LQ. Generally, there are three indicators used in the dynamic balance assessment of the lower limbs using the YBT-LQ, including extension distance in each direction, composite score, and extension distance difference. The extension distance difference refers to the difference in extension distance in the same direction between different legs, and always adopts the original value (cm). However, both the original value (cm) and the standard value (%) could be chosen to present the extension distance and the composite score. The standard value of the extension distance and the composite score could be normalized by calculating the quotient of the original value and leg length as shown in the following formula:

$$ExtDis_{\mathrm{std}}=\frac{OriDat}{Len_{LQ}}\times 100\%$$ (1)

$$ComSco_{\mathrm{std}}=\frac{\sum_{i=1}^{3}ExtDis_{i}}{3\times Len_{LQ}}\times 100\%$$ (2)

If the composite score is less than 95%, it indicates a high injury risk for the support leg. If the difference between the two sides is more than 5%, it indicates that the strength or balance of the left and right supporting legs are significantly different.

#### 2.2.3 Sports performance level of participants

In the research, the levels of sports performance (hereafter SP) of these subjects were divided into three categories: low, moderate, and high levels according to the following rules.

• low: refers to ordinary college students who have no professional physical training experience before their enrollment.
• moderate: refers to sport-specialized students who have undergone a period of sports training and passed the national sports entry examination.
• high: refers to elite athletes who were recruited from those students who have participated in national Wushu competitions and won at least one award.

#### 2.2.4 Physical activities level of participants

The International Physical Activity Questionnaire long form (hereafter IPAQ-LF) was adopted to assess and assort participants’ level of physical activity (PA), involving the energy consumption of four types of physical activities: occupation, transportation, household, and leisure on working days and weekends. It has been proved that the IPAQ has reasonable measurement properties for monitoring population levels of physical activity among 18–65 year old adults in diverse settings (Craig et al., 2003). As college students, subjects have not yet engaged in occupations, and due to the COVID-19 pandemic, all participants were restricted to engage in physical activities only on university campuses during the test procedures. The energy consumption data (MET min/week) of physical activity was calculated following the formulas as (Ding et al., 2021):

$$\begin{array}{ll} EngeryCon & =ActivityIntensity\times DailyActivityTime\\ & \;\times ExerciseDays \end{array}$$ (3)

The weekly PA of participants included three categories: vigorous PA (VPA) (i.e., aerobic exercises, heavy bearing, rapid running, etc.), moderate PA (MPA) (i.e., Tai chi, brisk walking, etc.), and light PA (LPA) (i.e., walking for leisure, sports at work or at home). All physical activities lasting less than 10 min continuously in a usual week reported in IPAQ-LF have been ignored and not included in the calculation. According to the study (Kim et al., 2018), 1 MET refers to the amount of oxygen consumed at rest, and VPA, MPA, and LPA can be expressed as 8 METs, 4 METs, and 3.3 METs, respectively (Ding et al., 2021). The metabolic equivalent of energy consumption of the participant’s physical activity was calculated following the formula:

$$\begin{array}{ll} MetabEquivalent & =EngeryCon_{\mathrm{V PA}}\times 8+EngeryCon_{\mathrm{MPA}}\\ & \;\times 4+EngeryCon_{\mathrm{LPA}}\times 3.3 \end{array}$$ (4)

According to the guidelines of the American College of Sports Medicine, total weekly physical activity which is beneficial to health should be more than 600 MET-minutes/week (Kojima, 2018). Based on the quantification of physical activity, the levels of sports performance of these subjects were divided into three levels: low, moderate, and high levels according to the following rules.

• low: refers to those participants whose metabolic equivalent of energy consumption of their weekly physical activities reported in the IPAQ-LF is less than 600 METs.
• moderate: refers to those participants whose metabolic equivalent of energy consumption of their weekly physical activities reported in the IPAQ-LF is more than 600 METs but less than 3,000 METs.
• high: refers to those participants whose metabolic equivalent of energy consumption of their weekly physical activities reported in the IPAQ-LF is more than 3,000 METs.

### 2.3 Procedure

At the beginning of the semester, participants performed the YBT-LQ test for dynamic lower limbs balance assessment. At the end of the semester, they filled out the questionnaires to provide some physiological information, such as gender, age, weight, height, and so on. Additionally, they were asked to report any sports injuries. All subjects were not in the stage of acute injury attack when they performed the YBT-LQ. After the professional instructor demonstrated the test actions, the subject first conducted warm-up activities, then completed acceptable test actions of the YBT-LQ three times in three different directions. At the same time, the observation values of test actions were synchronously recorded. If the quality of the test action failed to meet the requirements, the subject would be asked to redo the test action.

Participants completed an online questionnaire to self-report the intensity, frequency, and duration of their physical activities that occurred during the semester. All of these variables were used to calculate energy expenditure and rank the participant’s level of physical activity. All collected data were cleaned, sorted, and summarized. Furthermore, the study utilized a cross-sectional approach to conduct statistical analysis based on different levels of physical activity and sports performance. Additionally, a prospective study was conducted by comparing participants’ injury reports to the predicted sports injury risk based on the composite scores of the YBT-LQ.

### 2.4 Statistical analysis

To ensure the accuracy and completeness of the data analysis in the study, all collected data were cleaned, mainly focusing on removing and correcting dirty data. Non-essential symbols, such as superfluous spaces, and redundant or abnormal data, such as duplication and clerical mistakes, were removed or corrected. The data dimension and units were checked for consistency with the facts. Some missing values were found and supplemented after face-to-face inquiry and data recollection. The software Microsoft Excel 2021 and IBM SPSS for Windows version 26.0 were used for record storage and statistical data analyses. Based on the results of the data analysis, some calculation and visualization was carried out based on Python 3.9 and R 4.2 language and some necessary software package libraries.

To investigate whether the SP level and PA level influence the performance of YBT-LQ, a Spearman correlation analysis was conducted to explore the binary correlation between 13 factors, including the YBT-LQ score of the right and left leg, score difference of both legs, injury report, injury of ankle, knee, and hip, gender, body mass index (hereafter BMI), age, SP, and PA. The significance level was set at 0.01, and a two-tailed test was conducted to determine the statistical significance of the correlation. The statistical results were presented in the form of a thermodynamic diagram.

To distinguish college students with high sports injury risk from those with low risk through exploring a feasible optimal cutoff of YBT-LQ scores, the receiver operating characteristic (hereafter ROC) curve and the area under curve (hereafter AUC) were calculated with a 95% confidence interval (CI). The AUC displayed the measure of separability of classification presented by the ROC. When the value of AUC is greater than 0.7, the classification is acceptable. Better classification is achieved with higher values of the AUC. The ROC curves are plotted with a false-positive rate (hereafter FPR) against a true-positive rate (hereafter TPR), where FPR is on the *x*-axis and TPR is on the *y*-axis (Fawcett, 2006). The optimal cutoff value could be captured by identifying the point with the highest Youden Index (J index). The value of the Youden Index could be calculated using the following formula (B., 2019):

$$J_{max}={max}_{t}\left\{sensitivity\left(t\right)+specificity\left(t\right)-1\right\}$$ (5)

where the maximum Youden Index is reported, and t denotes the threshold of the binary classification.

## 3 Results

The results of data analysis were presented from three aspects: descriptive statistics including bioinformation and the distribution of the YBT-LQ scores, correlation analysis between 13 factors, and validation of the YBT-LQ as a predictor of sports injury based on dynamic lower limbs balance assessment with and without stratifying participants according to the levels of physical activity and sports performance.

### 3.1 Descriptive statistics

Descriptive statistics focused on physical conditions including population distribution, some bioinformation, injury rate, and YBT-LQ score distribution.

#### 3.1.1 Physical condition of participants

The details regarding the distributions of population, age, gender, height, weight, BMI, and metabolic equivalent (hereafter MET) are presented in Table 1. After population stratification according to different levels of sports performance, all participants (169 persons) were divided into three groups: low SP (61 persons, 36%), moderate SP (67 persons, 40%), and high SP (41 persons, 24%). Similarly, according to physical activity level, another three groups were: low PA (54 persons, 32%), moderate PA (71 persons, 42%), and high PA (44 persons, 26%). The study comprised 98 male and 71 female college students as participants. The gender ratio was nearly equivalent across all levels of physical activity and sports performance. The age range of participants was from 15 to 24 years old, and the age of the majority (62%) was 19 or 20 years old. The average and standard deviation of height, weight, BMI, and MET of all participants were respectively 172 cm ± 7.97, 65 kg ± 11.48, 22 *kg*\*m* ^2^±3.17 and 2450 ± 2581.46. Additionally, all participants exhibited right-sided limb dominance.

**TABLE 1:** Bioinformation of participants and distribution of population.

	Total	Age	Men	Height	Weight	BMI	MET
All	$169\left(42.01\%\right)$	20 ± 1.37	$98\left(57.99\%\right)$	172 ± 7.97	65 ± 11.48	22 ± 3.17	2450 ± 2581.46
LPA	$54\left(31.95\%\right)$	19 ± 1.23	$29\left(53.70\%\right)$	171 ± 7.68	64 ± 12.01	22 ± 3.68	421 ± 137.13
MPA	$71\left(42.01\%\right)$	19 ± 1.29	$42\left(59.15\%\right)$	173 ± 8.14	66 ± 11.90	21 ± 2.83	1781 ± 71.91
HPA	$44\left(26.03\%\right)$	20 ± 1.59	$27\left(61.36\%\right)$	172 ± 7.85	66 ± 9.93	22 ± 3.00	6089 ± 2404.36
LSP	$61\left(36.09\%\right)$	20 ± 0.87	$25\left(40.98\%\right)$	172 ± 8.49	66 ± 14.80	23 ± 4.38	468 ± 1736
MSP	$67\left(39.64\%\right)$	20 ± 1.13	$48\left(71.64\%\right)$	174 ± 7.42	66 ± 9.24	22 ± 2.18	2171 ± 1985.52
HSP	$41\left(24.26\%\right)$	19 ± 1.78	$25\left(60.98\%\right)$	170 ± 7.50	63 ± 8.42	22 ± 2.18	5260 ± 3875.32

Bioinformation statistics were performed after stratifying the population into different levels of physical activity and sports performance. Roughly a quarter of all participants achieved a high level of SP or PA. The weaker the level of SP, the greater the BMI value and the greater the probability of obesity. The same rules did not apply to those groups labeled with different PA levels. Those persons who reached a moderate level of PA presented better BMI than those who reached a low or high level of PA. As expected, participants’ MET value increased with higher levels of physical activity.

Table 2 provides a detailed account of the injury rate and population distribution of the entire sample, as well as of those who reported injuries in the ankle, knee, or hip, both before and after population stratification. During the semester, slightly over fifty percent of the participants reported at least one injury. The individuals labeled with a moderate physical activity level or a moderate sport performance level reported the highest incidence of injuries in their ankle, knee, or hip. Nearly all participants who reported injuries and were categorized with a low sport performance level experienced ankle injuries during the semester. As for injury ratio, participants labeled with low and high levels of PA and high levels of SP were more likely to report injuries.

**TABLE 2:** Distribution of population with injuries located in the ankle, knee, or hip before and after population stratification.

	InjuryRate	AnkleInjury $\left(Totall,Group\right)$	KneeInjury $\left(Totall,Group\right)$	HipInjury $\left(Totall,Group\right)$
All	$88\left(52.07\%\right)$	$83\left(49.11\%\right)$	$65\left(38.46\%\right)$	$6\left(3.55\%\right)$
LPA	$28\left(51.85\%\right)$	$21\left(12.43\%,38.89\%\right)$	$22\left(13.02\%,40.74\%\right)$	$1\left(0.59\%,1.85\%\right)$
MPA	$30\left(42.25\%\right)$	$36\left(21.30\%,50.70\%\right)$	$24\left(14.20\%,33.80\%\right)$	$3\left(1.78\%,4.23\%\right)$
HPA	$30\left(68.18\%\right)$	$26\left(15.38\%,59.09\%\right)$	$19\left(11.24\%,43.18\%\right)$	$2\left(1.18\%,4.55\%\right)$
LSP	$24\left(39.34\%\right)$	$23\left(13.61\%,37.70\%\right)$	$14\left(8.28\%,22.95\%\right)$	$1\left(0.59\%,1.64\%\right)$
MSP	$33\left(49.25\%\right)$	$30\left(17.75\%,44.78\%\right)$	$30\left(17.75\%,44.78\%\right)$	$3\left(1.78\%,4.48\%\right)$
HSP	$31\left(75.61\%\right)$	$24\left(14.20\%,58.54\%\right)$	$21\left(12.43\%,51.22\%\right)$	$2\left(1.18\%,4.88\%\right)$

#### 3.1.2 YBT-LQ scores of participants

**FIGURE 1:**
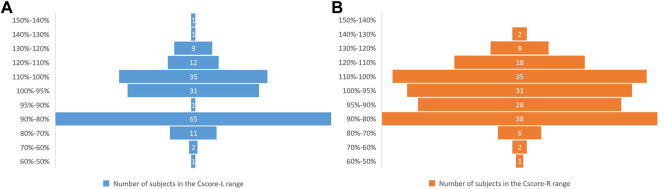
Distribution of population in different ranges of composite YBT-LQ score. **(A)** is the left leg **(B)** is the right leg.

Participants obtained composite scores of the YBT-LQ ranging from 58% to 141% for left leg support and from 54% to 138% for right leg support. With a 95% threshold for composite scores on both support legs during the YBT-LQ assessment, 94 (55%) participants passed the left leg assessment, and 89 (52%) passed the right leg assessment. Figure 1 shows the distribution of composite scores of all participants in the YBT-LQ assessment at 10 intervals. Specifically, Figure 1A depicts the composite scores for the left leg as the supporting leg during the test, while Figure 1B displays the composite scores for the right leg as the supporting leg. As presented in Figure 1, it can be concluded that the majority of participants performed better when using their right leg as the supporting leg compared to the left leg.

The difference between the composite scores of the left and right leg indicated the dynamic balance gap on both sides of the body. In general, a high injury risk is defined as a difference of 5% or more in composite scores between the two sides. Using a composite score threshold greater than 95%, the statistics result revealed that 82 persons (48%) passed both sides YBT-LQ assessments, 19 persons (11%) passed only one side YBT-LQ assessment, and 68 persons (41%) failed both sides YBT-LQ assessment. The largest difference in composite scores between the left and right leg was 26.7%, with composite scores of 89.9% and 116.6% for the left and right legs, respectively. There were a total of 43 (25%) persons whose differences between the composite scores of the left and right leg were more than 5%.

### 3.2 Factor correlation analysis

**FIGURE 2:**
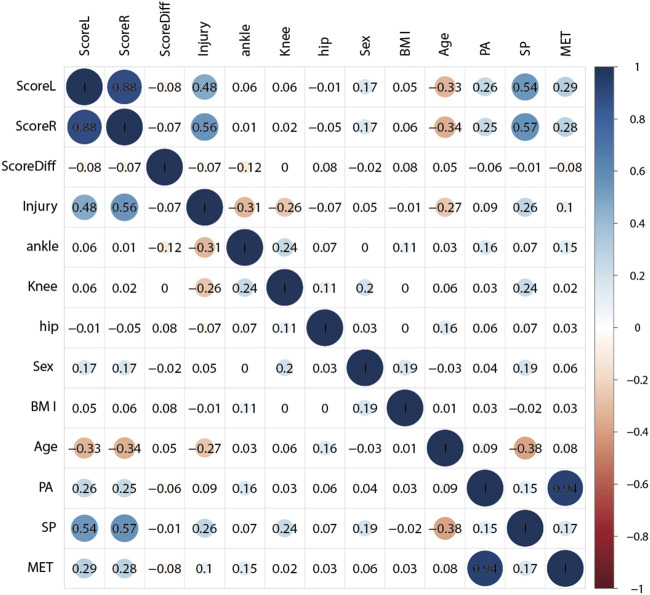
Heatmap of the 13 factors correlation analysis results.

In the study, a total of 13 factors, including the composite score of the left leg (ScoreL) and the right leg (ScoreR), the difference of the above two composite scores (SocreDiff), all types of reported injuries, reported ankle injury, reported knee injury, reported hip injury, gender, BMI, age, the level of PA, the level of SP, and MET, were subjected to factor correlation analysis. Figure 2 presents the numerical value of correlation coefficient and visualization of factors correlations analysis result. When the significance level of the test was set at 0.01, the results showed a significant correlation between the composite score (both ScoreL and ScoreR) of the YBT-LQ and injury reports, age, PA level, SP level, and MET. Generally speaking, a correlation coefficient absolute value falling between 0.3 and 0.5 indicates a moderate level of correlation, while one greater than 0.5 indicates a strong correlation. Therefore, the composite scores (ScoreL and ScoreR) showed a strong correlation with SP level and injury reports, a moderate correlation with PA level (almost), MET (almost), and age (negative), and a weak correlation with BMI and ScoreDiff (negative). There was a strong correlation between ScoreL and ScoreR. Furthermore, a moderate correlation was observed between injury reports and age (negative), as well as SP level.

### 3.3 ROC curve and AUC analyses

To explore the effectiveness of dynamic lower limbs balance assessment using the YBT-LQ, the ROC curve was calculated utilizing records of participants’ YBT-LQ scores and injury reports to identify an optimal cut-off value that can support an early warning of sports injury risk among Chinese physical education college students.

#### 3.3.1 Before population stratification

**FIGURE 3:**
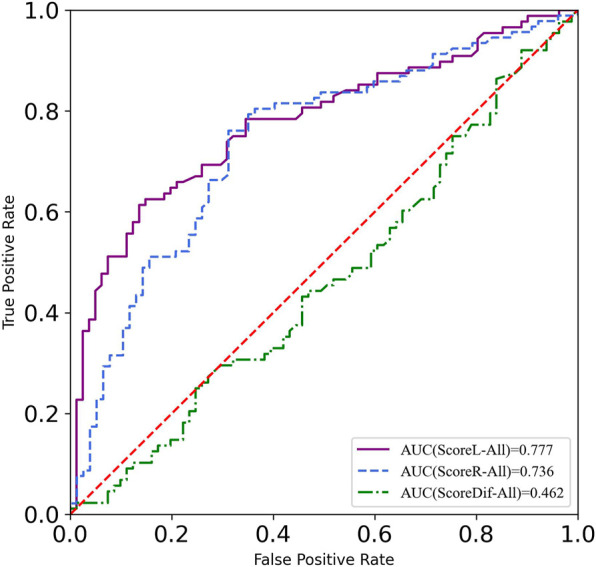
ROC of left leg, right leg, and difference of YBT scores before population stratification.

The calculations of the ROC curves were carried out, utilizing the reported injury data of all participants with ScoreL, ScoreR, and ScoreDiff as input variables. The visualization of the three total calculation results was integrated and is shown in Figure 3. The AUC results of the ROC curves were 0.777 for ScoreL, 0.823 for ScoreR, and 0.462 for ScoreDiff. These values indicated that dynamic lower limb balance assessment based on the composite scores of YBT-LQ proved to be a valid method for predicting injury risk, as well as the difference between the two composite scores being invalid as a predictor of sport risk injury. The study also indicated that the composite scores of the right leg as the standing leg showed better efficiency in predicting sports injuries than those of the left leg.

To figure out the optimal cut-off value of the YBT-LQ for early warning of sports injury risk, the highest Youden Index (or Youden’s J Statistic) should be found. The thresholds of the composite score of the left leg (ScoreL) and the right leg (ScoreR) were 97.8% and 95.2%, respectively.

#### 3.3.2 After population stratification

**FIGURE 4:**
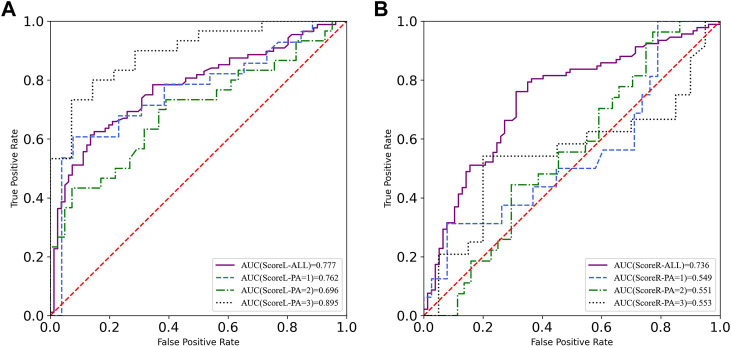
ROC curves of YBT-LQ scores according to different levels of PA. **(A)** is the left leg **(B)** is the right leg.

**FIGURE 5:**
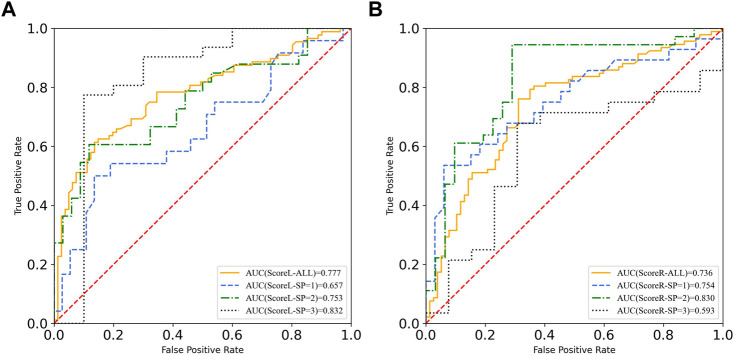
ROC curves of YBT-LQ scores according to different levels of SP. **(A)** is the left leg **(B)** is the right leg.

Concerning the strong correlation between the composite score of the YBT-LQ and SP level, the moderate correlation between the composite score of the YBT-LQ and PA level, the ROC was calculated after participants stratification according to PA level and SP level, respectively. The visualization of a total of 14 calculation results were integrated and are shown in Figures 4, 5.

After participant stratification, ROC curves labeled with different levels of PA were calculated. The AUC results proved that the efficiency of sports injury prediction based on the composite score of the YBT-LQ changed. Specifically, the AUC of ScoreL labeled with the high level of PA was nearly 0.9, which meant that those participants with reported injuries could be distinguished excellently from those participants without sports injuries in the population labeled with the high PA level. However, the AUC results of the composite YBT-LQ score of the right leg after participant stratification according to different levels of PA were not excellent. Therefore, the AUC results of the group with different levels of PA presented acceptable ROC values, which means the composite YBT-LQ score of the left leg was up to be a predictor of sports injury through assessment of dynamic lower limbs balance, especially in the population with the high level of PA.

Figure 5 presents the AUC results of composite scores of the YBT-LQ based on the population stratification according to the different levels of SP. Similar to the ScoreL of the YBT-LQ according to the different levels of PA, the AUC results of composite YBT-LQ scores of the left leg proved the validity of it as a predictor of sports injury risk because of the acceptable values of the ROC (all more than 0.7), especially in the population with a high SP level.

**FIGURE 6:**
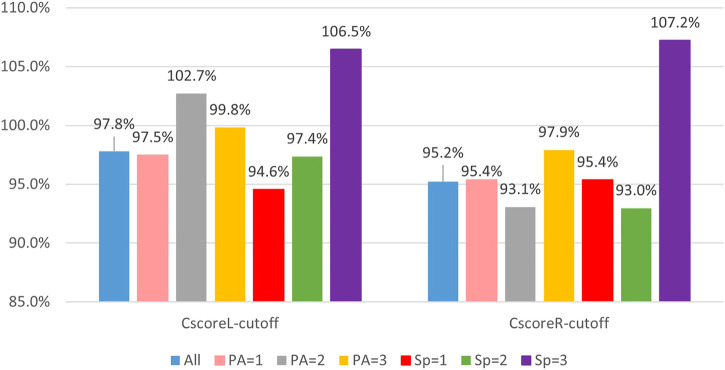
Cutoff composite score of the YBT-LQ according to different levels of PA or SP.

All cutoff values of composite scores of the YBT-LQ were more or less than 95%, as presented in Figure 6, and different from each other. The above result discovered that the physical activity level and sports performance level indeed influenced the efficiency of the YBT-LQ for sports injury prediction.

## 4 Discussion

Dynamic balance ability is essential for sports injury prevention because it helps humans maintain control over their body movements, which reduces the risk of falls, twists, and other movements that can lead to injury. The mechanism of maintaining human balance is very complex. From the perspective of physiology, human balance control mainly depends on the coordination of the central nervous system and three elements, including the vestibular system, proprioception system, and visual system. The vestibular system plays the most important role in maintaining balance, which is born naturally and built at baby age. The mechanisms of age, muscle strength, gender, BMI, and physical exercise affecting dynamic balance have always been a hot research issue. With age advancement, physiological changes in sensory, somatosensory, and motor systems often lead to a reduced dynamic balance control ability (Startzell et al., 2015) (Buková et al., 2023). In addition, muscle strength is proven to be associated with dynamic balance in healthy adults, such as isometric force control (Mear et al., 2023). There are many related studies on the relation between gender and human balance ability, but no consistent conclusion has been reached. Some people think that men have stronger muscle strength and can trigger better balance feedback than women, while others think that women have a lower center of gravity and better flexibility, so women have better balance ability (Ren and Chen, 2021). Height, weight, and BMI were proven to be negatively correlated with balance ability. A larger weight may affect brain agility, make actions and reactions hesitant, and lead to the decline of body balance ability. Height can affect an individual’s center of gravity. The higher the center of gravity, the more challenging it can be to maintain balance. Research shows that physical exercise can effectively enhance and improve human balance control, such as simplified Taijiquan (Hu and Ma, 2015). The level of physical activity (PA) and sports performance (SP) is closely related to muscle strength, BMI, and physical exercise; however, it is unclear that whether PA and SP can influence the accuracy of dynamic lower limb balance assessment. The study aimed to investigate whether physical activity level and sports performance level have an impact on dynamic lower limb balance ability and to validate the effectiveness of the YBT-LQ in preventing sports injuries among Chinese physical education college students.

The Star Excursion Balance Test (SEBT) is one of the most common methods to measure the control ability of dynamic posture for athletes or patients (Gribble et al., 2012b). The Y-Balance Test (YBT) was developed based on a modified SEBT and is used to assess the stability of limbs, future risk of injury, functional insufficiency after injury, and to monitor the rehabilitation process. The YBT is a reliable and valid tool for assessing dynamic balance and neuromuscular control and has been shown to be useful in identifying individuals at risk for lower extremity injury (Plisky et al., 2006). The performance of the YBT is affected by a variety of factors, mostly biological and kinematic. Examples of biological factors include age, gender, and strength, while kinematic factors include sports events, training methods, and competition level. Therefore, the norm data is suggested to be used to improve the assessment of dynamic balance and predict injury disk based on the classification of different sports populations in the research (Su et al., 2021). In order to reduce the variability for accurate assessment of postural balance, anthropometric characteristics, gender, and lower limb strength, having a different impact on the YBT measurement, should be controlled (Fusco et al., 2020). Based on the aforementioned explanation, the study selected the YBT-LQ as a tool for dynamic lower limbs balance assessment and standardized the processes through subject selection, protocol consistency operation, data cleaning, analysis, and processing standardization.

There were a total of 169 voluntary participants enrolled in the study. They provided some anthropometric characteristics and other bioinformatic data online. They completed the YBT-LQ at the beginning of the semester and reported sports injury conditions at the end of the semester. All subjects of the study were from one Chinese physical education college and consisted of ordinary students without professional physical training experience, sport-specialized students with more than a year of training experience, and elite athletes who were awarded at least once in national Wushu competitions. The participants in the study were limited to similar aged (SD:1.37) college students with gender in moderate distribution. Although the limb dominance of participants was all the right side, limb dominance has been proven to have no effect on YBT performance (Stoddard et al., 2022).

A pairwise correlation analysis between the composite scores of the YBT-LQ and some factors affecting the dynamic lower limbs balance were conducted. The study found a strong correlation between YBT-LQ composite scores and both sports performance (SP) level and sports injuries. Moderate correlations were also observed between YBT-LQ composite scores and PA level (almost), age (negative), and metabolic equivalent (MET) (almost). The negative correlation between YBT-LQ composite scores and age could be due to the fact that elite athletes were typically younger than regular and sport-specialized students. They also tended to have higher levels of sports performance (SP) and physical activity (PA), which results in better YBT-LQ performance. Factor correlation analysis in the study showed that the physical activity level and sports performance level indeed influenced the composite scores of the YBT-LQ.

To further investigate whether and how PA and SP impacted the sports injury prediction based on dynamic lower limbs balance assessment, the participants were stratified according to different levels of PA and SP. The threshold of the composite score of the YBT-LQ is generally 95% for injury risk warning. However, some studies provided different thresholds for sports injury warning. YBT-UQ composite scores ≤81.1% predicted upper quadrant musculoskeletal injury in military personnel over a 12-month follow-up while controlling for age and sex (Campbell et al., 2022). A cutoff point of 89.6% composite score on the SEBT was obtained for non-contact lower extremity injury prediction in American college football players (Butler et al., 2013). The results in the study presented the cutoff value of the composite scores of the YBT-LQ as quite different from 95% both before and after participant stratification according to the PA level and SP level. The difference between the two composite scores presented weak or even no correlation with other factors affecting dynamic balance ability in the pairwise correlation analysis. Therefore, only composite scores of the YBT-LQ were adopted as the indicator in sports injury prediction. The ROC curves and AUC were calculated in the study to explore the efficiency of the YBT-LQ composite scores in sports injury risk prevention. Before participant stratification, the AUC value of the ROC curves proved that composite scores of the YBT-LQ were eligible as a predictor of sports injury risk based on the dynamic lower limbs balance assessment. After participant stratification according to the PA level and SP level, respectively, the AUC values of the ROC curves labels with different levels changed. The cutoff values of the composite scores of the YBT-LQ varied widely and all of them were either slightly above or below the general threshold of 95% for sports injury warning. No matter whether the standing leg was the left leg or the right leg during the test, the cutoff values of the composite scores of the YBT-LQ of population with a high level of SP were more than 105%.

In summary, population stratification according to different levels of PA and SP is necessary to accurately evaluate the dynamic lower limbs balance ability using the YBT-LQ for sports injury prevention. However, the age range of the study participants was limited to 15–22 years old. Further exploration is needed to determine whether the research conclusion can be applied to other age groups. This is one of the directions of our future research. At the same time, our future study will also focus on exploring the underlying mechanisms by which different levels of physical activity and sports performance affect sports injury prediction.

## 5 Conclusion

To improve health by avoiding sports injuries during exercises, some assessments can be conducted in advance. Dynamic balance ability is closely related to sports injuries. The YBT-LQ is a widely used dynamic lower limbs balance assessment tool; however, it is unclear whether and how physical activity and sports performance influence the performance of the YBT-LQ. The study can be concluded with three points. Firstly, the performance of the YBT-LQ was proven to correlate with the levels of physical activity and sports performance. Secondly, the composite scores of the YBT-LQ provided an acceptable efficiency of prediction of sports injury risk based on dynamic lower limbs balance assessment in the population of Chinese physical education college students. Lastly, those persons who have obtained a high sports performance, such as elite athletes, should be assessed singly through being distinguished from ordinary college students because of a high optimal cutoff of the composite score of the YBT-LQ in sports injury prediction. The study has practical significance for improving the utilization of the YBT-LQ in assessing the dynamic balance of the lower limbs.

## Data Availability

The raw data supporting the conclusion of this article will be made available by the authors, without undue reservation.
